# Insulin-like growth factor binding protein-3 inhibits cell adhesion via suppression of integrin β_4_ expression

**DOI:** 10.18632/oncotarget.3825

**Published:** 2015-04-14

**Authors:** Hyo-Jong Lee, Ji-Sun Lee, Su Jung Hwang, Ho-Young Lee

**Affiliations:** ^1^ College of Pharmacy, Inje University, Gimhae, Gyungnam, Republic of Korea; ^2^ u-Healthcare and Anti-aging Research Center (u-HARC), Inje University, Gimhae, Republic of Korea; ^3^ Biohealth Products Research Center (BPRC), Inje University, Gimhae, Republic of Korea; ^4^ College of Pharmacy and Research Institute of Pharmaceutical Sciences, Seoul National University, Seoul, Republic of Korea

**Keywords:** IGFBP-3, IGF-1, integrin, cell adhesion, AP-1, angiogenesis

## Abstract

We previously reported that IGF binding protein-3 (IGFBP-3), a major IGF-binding protein in human serum, regulates angiogenic activities of human head and neck squamous cell carcinoma (HNSCC) cells and human umbilical vein endothelial cells (HUVECs) through IGF-dependent and IGF-independent mechanisms. However, the role of IGFBP-3 in cell adhesion is largely unknown. We demonstrate here that IGFBP-3 inhibits the adhesion of HNSCC cells and HUVECs to the extracellular matrix (ECM). IGFBP-3 reduced transcription of a variety of integrins, especially integrin β_4_, and suppressed phosphorylation of focal adhesion kinase (FAK) and Src in these cells through both IGF-dependent and IGF-independent pathways. IGFBP-3 was found to suppress the transcription of c-fos and c-jun and the activity of AP1 transcription factor. The regulatory effect of IGFBP-3 on integrin β_4_ transcription was attenuated by blocking c-jun and c-fos gene expression *via* siRNA transfection. Taken together, our data show that IGFBP-3 has IGF-dependent and -independent inhibitory effects on intracellular adhesion signaling in HNSCC and HUVECs through its ability to block c-jun and c-fos transcription and thus AP-1-mediated integrin β_4_ transcription. Collectively, our data suggest that IGFPB-3 may be an effective cancer therapeutic agent by blocking integrin-mediated adhesive activity of tumor and vascular endothelial cells.

## INTRODUCTION

The integrin superfamily is a major class of cell surface receptors for ECM molecules. Members of this superfamily are composed of α and β subunits. At least 24 different heterodimers with distinct tissue distributions and overlapping ligand specificities can be formed by various combinations of the 18 α and 8 β subunits [1]. Endothelial cells express a variety of integrins, including α_5_β_1_, α_v_β_1_ and α_v_β_5_, which are receptors for fibronectin (FN); α_3_β_1_, α_6_β_1_, and α_6_β_4_, which are receptors for laminin; and α_v_β_3_, which is a receptor for vitronectin, osteopontin, and collagen [2]. Integrins play an important role in cell-cell and cell-matrix adhesion and thus are involved in tumor growth and metastasis through numerous cellular functions, including cell migration, invasion, and extravasation [3]. Integrin-mediated signaling mechanisms typically include the activation of FAK and Src, which leads to the organization of the actin cytoskeleton at sites of focal adhesion to the ECM [3] and remodeling of the adhesion complex [2, 4]. Consequently, targeting integrin-mediated signaling may represent a powerful anticancer therapy [5].

IGFBP-3 is a member of a family of 6 IGFBPs and regulates the interaction between IGF-I and IGF-II and their receptor (IGF-IR) [6]. IGFBP-3 is a major IGF-binding protein in adult serum, and its synthesis and secretion vary by cell type and species of origin [6, 7]. The antitumor activities and mechanisms of action of IGFBP-3 have been extensively validated in various preclinical model systems [6, 8, 9]. We and others have demonstrated that IGFBP-3 is a potent inducer of apoptosis in a variety of human cancer cell types by inhibiting IGF-mediated signaling pathways [10-12]. IGFBP-3 also induces IGF-independent antiproliferative activities in IGF-IR null fibroblasts [13] and breast [14] and prostate cancer cells [15, 16]. We have also demonstrated that IGFBP-3 exhibits potent IGF-independent antiangiogenic activities in non-small cell lung cancer (NSCLC) cells, HNSCC cells, and HUVECs *in vitro* and *in vivo* [6, 8, 9]. Although we have consistently observed the suppression of migration and invasion of these cell types by IGFBP-3, the effects of IGFBP-3 on cell-to-matrix adhesion are largely unknown.

This study sought to investigate the role of IGFBP-3 in the adhesion of cancer and vascular endothelial cells to the ECM and the underlying molecular mechanism, with a focus on IGF-1 dependency. Our findings suggest that IGFBP-3 inhibits the adhesion of both HNSCC cells and HUVECs to the ECM at least in part by negatively regulating the expression of integrin β_4_ in an IGF-dependent and IGF-independent manner. These data further explain how IGFBP-3 regulates cancer cell metastasis and tumor angiogenesis.

## RESULTS

### IGFBP-3 mediates cell-to-matrix adhesion of UMSCC38 cells and HUVECs

**Figure 1:**
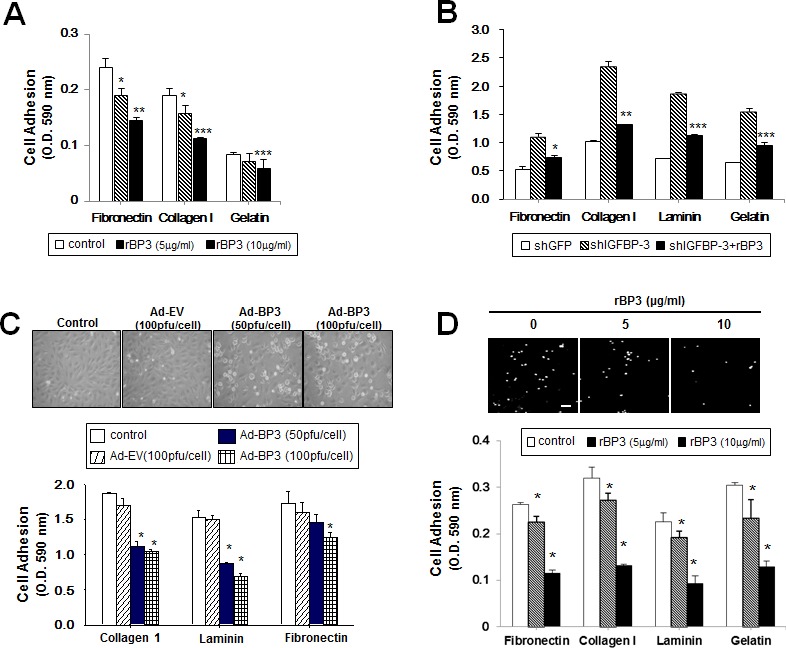
IGFBP-3 inhibits cell-to-matrix adhesion of UMSCC38 cells and HUVECs **A.** Cell-to-matrix adhesion was assayed using UMSCC38 cells treated with different doses of recombinant human IGFBP-3 (rBP3). Cell adhesion values are expressed relative to the adhesion of untreated cells, normalized to 100%. The error bar represents the S.D.; *, *P* < 0.05; **, *P* < 0.01; ***, *P* < 0.001. **B.** Cell-to-matrix adhesion assay using UMSCC38 cells stably transfected with retroviral pSM2 plasmids [control shGFP RNA (shGFP) or the shIGFBP-3 RNA (shIGFBP-3)]. The error bar represents the S.D.; *, *P* < 0.05; **, *P* < 0.01; ***, *P* < 0.001. Western blotting (top) analysis of IGFBP-3 protein levels in UMSCC38 stable cell lines was performed. **C.** Cell-to-matrix adhesion assay using HUVECs infected with either Ad-EV or Ad-BP3 as indicated. Each sample was assayed in triplicate, and the experiment was repeated three times independently. *, *P* < 0.05 compared with Ad5CMV. Representative images (top) indicate the morphology of infected HUVECs. **D.** Cell-to-matrix adhesion assay using HUVECs treated with different doses of rBP3. The error bar represents the S.D.; *, *P* < 0.05; #, *P* < 0.01 compared with the control. HUVECs labeled with Hoechst were seeded onto a gelatin-coated 96-well plate for 15 min. White dots indicate Hoechst-labeled HUVECs binding to the gelatin (top). Scale bar = 50 μm.

We have reported that induction of IGFBP-3 expression by adenoviral infection or by treatment with rBP3 or SCH66336 (a farnesyl transferase inhibitor) suppresses the activities of growth, angiogenesis, and metastasis in NSCLC and HNSCC cells [17, 18]. To further study the effects of IGFBP-3 on tumor growth and progression, we investigated whether IGFBP-3 can alter cancer cell adhesion to ECM. To this end, we treated UMSCC38 HNSCC cells with rBP3. As shown in Figure 1A, rBP3 markedly reduced cell adhesion to fibronectin, type I collagen, and gelatin in a dose-dependent manner. Next, we used UMSCC38 cells stably transfected with either control (shGFP) or IGFBP-3 shRNA (shIGFBP-3) to confirm the regulatory role of IGFBP-3. UMSCC38 cells expressing shIGFBP-3 exhibited increased binding to fibronectin, type I collagen, laminin, and gelatin compared with shGFP-expressing cells; this increased binding was reversed by rBP3 treatment (Figure 1B). Because adhesion of vascular endothelial cells (ECs) within the tumor microenvironment plays a fundamental role in tumor angiogenesis and progression [8], we examined the effect of IGFBP-3 on HUVEC adhesion to ECM using HUVECs that were infected with either Ad-EV or Ad-BP3. Ad-BP3-infected HUVECs were rounded, and their spreading on gelatin-coated plates was inhibited in a dose-dependent manner (Figure 1C, top). Furthermore, Ad-BP3-infected HUVECs showed decreased binding to type I collagen, laminin, and fibronectin compared with Ad-EV-treated HUVECs (Figure 1C). Consistent with the results in UMSCC38 cells, the exogenous addition of rBP3 also resulted in a dose-dependent inhibitory effect on HUVEC adhesion to matrix proteins (Figure 1D). Representative data demonstrating the effects of rBP3 on HUVEC adhesion to gelatin is presented in Figure 1D top. The inhibitory effect of 10 μg/ml rBP3 on binding to fibronectin, type I collagen, laminin and gelatin cell-to-matrix was 43.9%, 41.0%, 41.2%, and 42.1%, respectively. We observed that viability of UMSCC38 cells was significantly affected neither by recombinant IGFBP-3 treatment nor by shIGFBP-3 transfection (Supplementary Figure 1). Therefore, it was likely that IGFBP-3 has inhibitory effects on cell adhesion independent of its effects on cell viability.

### IGFBP-3 decreases integrin β_4_ expression and inactivates downstream FAK/Src in UMSCC38 cells and HUVECs

**Figure 2:**
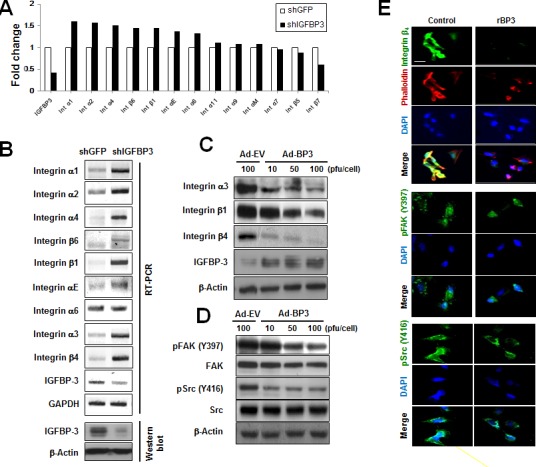
IGFBP-3 decreases integrin β expression and FAK activation in UMSCC38 cells and HUVECs **A.** The fold changes in the mRNA transcripts in the indicated UMSCC38 cell lines were determined using microarrays as described in the Material and Methods. The expression of integrins and IGFBP-3 was analyzed, and expression levels are expressed relative to shGFP-transfected cells, normalized to 100%. **B.** RT-PCR analysis confirmed integrin mRNA expression in UMSCC38 cells infected with either shGFP RNA (shGFP) or shIGFBP-3 RNA (shIGFBP-3) (top). Western blotting for IGFBP-3 protein level in UMSCC38 cells infected with either shGFP or shIGFBP-3 (bottom). **C.** Western blotting for integrin protein levels in HUVECs infected with either Ad-EV or Ad-BP3 as indicated. **D.** Western blotting analysis of the indicated proteins in HUVECs infected with the indicated titer of Ad-EV or Ad-BP3 for 2 days. Cell lysates were subjected to western blot analysis using antibodies against FAK, Src, and their phosphorylated forms. **E.** Immunofluorescent staining of integrin β_4_ (green) and phalloidin (red, top); phospho-FAK-397Y (green, middle); phospho-Src-416Y (green, bottom) in HUVECs. Nuclei (blue) were counterstained with DAPI (4′,6-diamidino-2-phenylindole). Scale bar is 20 μm.

To investigate the mechanism by which IGFBP-3 regulates cell adhesion, we examined the effects of IGFBP-3 on a panel of cell-adhesion-associated genes using UMSCC38 cells stably transfected with shGFP or shIGFBP-3. A microarray analysis revealed that integrins α_1_, α_2_, α_4_, β_6_, β_1_, α_E_, and α_6_ were affected by IGFBP-3 (Figure 2A). We further performed RT-PCR analysis to confirm the effects of IGFBP-3 on integrins expression. Because the IGFBP-3 suppressed UMSCC38 cell adhesion to laminin with a great potency, we also analyzed the effects of IGFBP-3 on the expression of integrin α_3_ and β_4_, which are receptors for laminin. Consistent with the microarray data, we observed that UMSCC38 cells expressing shIGFBP-3 exhibited upregulation of large numbers of integrins (including α_1_, α_2_, α_4_, β_6_, β_1_, α_E_, α_6_, α_3_ and β_4_) compared to cells expressing shGFP. In particular, the expression of integrins α_3_, β_1_ and β_4_ increased significantly upon downregulation of IGFBP-3 (Figure 2B). We further confirmed the effects of IGFBP-3 on the expression of integrins in HUVECs by performing a gain-of-function study using HUVECs that were infected with either empty adenoviruses (Ad-EV) or IGFBP-3-expressing adenoviruses (Ad-BP3). Ad-BP3 caused dose-dependent decreases in integrin α_3_, β_1_ and β_4_ expression, particularly integrin β_4_, in HUVECs (Figure 2C). It is reported that integrin β_4_ is upregulated in both angiogenic endothelial cells and tumor cells, facilitating angiogenesis [19]. Also, integrin β_4_ interacts with multiple receptor tyrosine kinases, such as EGF-R, ErbB2, and Met and enhances the signaling function of RTKs, in which deregulated joint β_4_-RTK signaling influences tumor progression [20, 21]. Based on these evidences and our results, we focused the integrin β_4_ as an attractive target for anti-angiogenesis and cancer therapy and examined the effect of IGFBP-3 on integrin β_4_.

Cytoskeletal organization and adhesion are controlled by complex coordination of focal adhesions, which is a hallmark of integrin interaction with ligands [22]. Integrins can initiate signaling cascades upon activation by the recruitment and activation of signaling proteins, such as the non-receptor tyrosine kinases FAK and c-Src, thereby forming a kinase complex [4]. After activation, the complex can phosphorylate a vast array of adaptor proteins, including p130C and paxillin, which can activate downstream Akt and Erk, thereby regulating cell motility and adhesion as well as cell survival and proliferation [23, 24]. We then measured the effects of rBP3 on FAK and Src phosphorylation. Western blotting revealed that infection with Ad-BP3 decreased both pFAK (Y397) and pSrc (Y416) levels in HUVECs. (Figure 2D). Immunofluorescence staining showed co-localization of Alexa 594-labeled phalloidin (a marker of actin filaments in the cytoskeleton, red) and integrin β_4_ (green) in HUVECs. Treatment with rBP3 induced microscopically characterized alterations in cell adhesion plaques and cytoskeletal assembly along with a decrease in phalloidin expression, pFAK (Y397), and pSrc (Y416) levels in HUVECs (Figure 2E). Collectively, these results suggest that IGFBP-3 suppresses integrin α_3_, β_1_ and β_4_ expression, resulting in dephosphorylation of FAK and Src and actin cytoskeletal reorganization in HUVECs.

### IGFBP-3 inhibits integrin β_4_ in both IGF-dependent and IGF-independent manner

**Figure 3:**
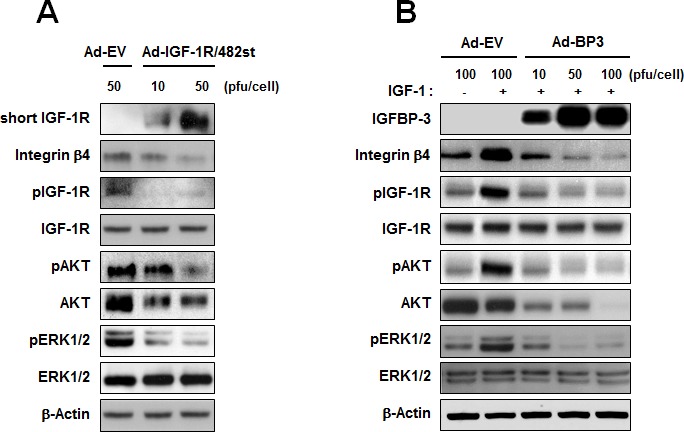
IGFBP-3 inhibits IGF-mediated integrin β expression in HUVECs **A.** Western blotting analysis of the expression of integrin β_4_, phosphorylated IGF-1R (p-IGF-1R), IGF-1R, phosphorylated AKT (pAKT), AKT, phosphorylated ERK1/2 (pERK1/2), and ERK1/2 in HUVECs infected with either Ad-EV or Ad-IGF-1R/482 (dominant negative soluble form of IGF-1R). **B.** Western blotting analysis of the indicated proteins in HUVECs infected with the indicated titer of Ad5CMV or Ad5CMV-BP3 for 2 days and stimulated with IGF-1 (100 ng/ml) for 15 min.

It has been reported that IGF-I induces integrin expression and migratory activity in chondrosarcoma cells [25] and epithelial cells [26]. To investigate whether the effects of IGFBP-3 on integrin expression and downstream signaling are IGF-dependent in HUVECs, we determined whether the IGF-1R pathway induces integrin expression and, if so, whether IGFBP-3 exerts regulatory actions on IGF-1-mediated integrin expression in HUVECs. We first assessed the role of IGF signaling in integrin β_4_ expression in HUVECs. IGF signaling was blocked by infection with adenoviruses expressing the dominant negative soluble form of IGF-1R (Ad-IGF-1R/482st). As expected, a viral dose-dependent increase in the expression of the truncated receptor was observed (Figure 3A). Ad-IGF-1R/482st also markedly decreased the expression of integrin β_4_ as well as the phosphorylated forms of IGF-1R, FAK, AKT, and ERK1/2 in HUVECs. We then incubated HUVECs with IGF-1 (100 ng/ml) in the presence or absence of Ad-EV or Ad-BP3. IGF-1 clearly increased the expression of integrin β_4_, whereas the overexpression of IGFBP-3 by Ad-BP3 prevented the IGF-induced increases in integrin β_4_, pIGF-1R, pAKT, and pERK1/2 expression (Figure 3B). We observed that total AKT expression was also decreased by Ad-IGF1R/482st or Ad-BP3. Hence, we further determined whether blockade of IGF1R by transfection with siRNA or treatment with recombinant BP3 also decrease total AKT expression. We found that IGF1R-specific siRNA or recombinant BP3 decreased integrin beta 4 without affecting total-AKT expression (Supplementary Figure 2A and 2C). Therefore, it was likely that adenoviral infection caused an artificial decrease in Akt expression by subverting protein expression machinery. Neverthless, these findings suggest that IGFBP-3 inhibits intracellular cell adhesion signaling by regulating the expression of a subset of integrins, especially integrin β_4_, through IGF-dependent mechanisms.

**Figure 4:**
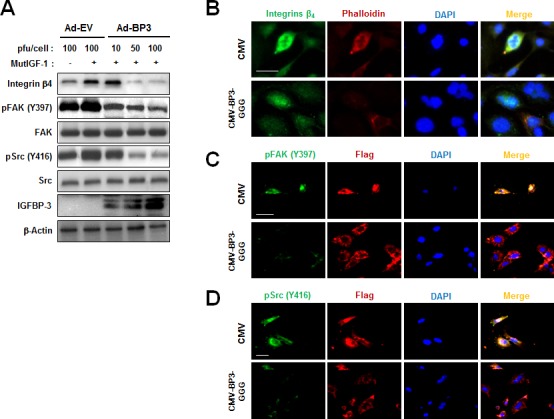
IGFBP-3 represses integrin β expression in an IGF-independent manner in HUVECs **A.** Western blotting analysis of the indicated proteins in HUVECs infected with the indicated titer of Ad-EV or Ad-BP3 for 2 days and stimulated with 100 ng/ml mutant recombinant IGF-1 (MutIGF-2; resistant to IGFBP-3 for 25 min) for 15 min. Whole-cell lysates isolated from the indicated HUVECs were subjected to western blot analysis of integrin β_4_, phosphorylated tyrosine residue 397 FAK [pFAK(Y397)], total-FAK, phosphorylated tyrosine residue 416 Src (pSrc(Y416)), total-Src and IGFBP-3. **B.** Immunofluorescent staining of integrin β_4_ (green), phalloidin (red) in HUVECs transfected with either pCMV6-empty vector (CMV) or pCMV6-IGFBP-3-GGG (CMV-BP3-GGG). **C.** Immunofluorescent staining of FLAG (red) and pFAK(Y397) (green). **D.** Immunofluorescent staining of FLAG (red) and anti-pSrc(Y416) (green). Nuclei (blue) were counterstained with DAPI. Scale bars are 20 μm.

We next assessed whether IGF-independent mechanisms were involved in the IGFBP-3-mediated regulation of integrin expression by using two different mutant systems: a recombinant IGF-1 mutant that is unable to bind IGFBP-3, and IGFBP-3-GGG, which contains a substitution mutation in the IGF-binding domain [8]. HUVECs were unstimulated or stimulated with recombinant mutant IGF-1 (MutIGF-1) in the absence or presence of Ad-EV or Ad-BP3. MutIGF-1 induced integrin β_4_ protein expression in association with FAK and Src phosphorylation, all of which were effectively suppressed by Ad-BP3 infection (Figure 4A). To confirm the IGF-independent action of IGFBP-3, we transiently transfected cells with either control pCMV6-Flag (CMV) or pCMV6-Flag-IGFBP-3-GGG (CMV-BP3-GGG), a mutant form of IGFBP-3 in which three residues important for the IGF binding domain were mutated to glycine (Gly^56^, Gly^80^ and Gly^81^) [27]. As shown in the results from the immunofluorescence staining, the IGFBP-3 mutant (CMV-BP3-GGG) reduced integrin β_4_ expression and cytoskeletal assembly along with phalloidin staining (Figure 4B). To further confirm the effects of mutant IGFBP-3, we analyzed the changes in FAK and Src phosphorylation. Similar to wild type IGFBP-3, mutant IGFBP-3 expression resulted in reduced expression of pFAK and pSrc (Figure 4C and 4D). Taken together, these data suggest that the inhibitory effects of IGFBP-3 on integrin β_4_ expression and intracellular effectors of cell adhesion signaling occur *via* IGF-independent mechanisms.

### IGFBP-3 inhibits integrin β_4_ via down-regulation of AP-1

**Figure 5:**
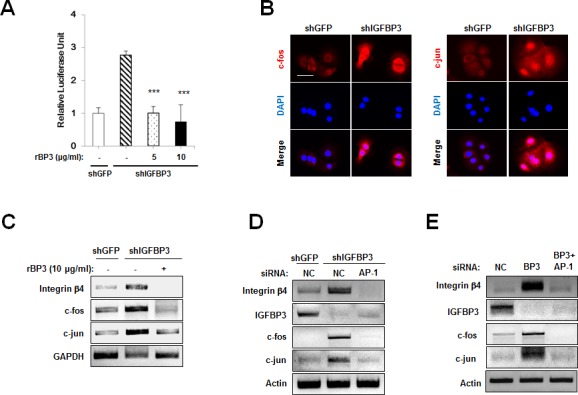
IGFBP-3 reduces integrin β expression in UMSCC38 cells and HUVECs via AP-1 downregulation **A.** Luciferase reporter assay in UMSCC38 cells stably transfected with retroviral pSM2 plasmids (either control shGFP RNA (shGFP) or the shIGFBP-3 RNA (shIGFBP-3)). After co-transfection with the vector/AP-1 promoter reporter, rBP3 was treated as indicated. Relative luciferase units are presented as the mean ± SEM of three independent experiments. **B.** Immunofluorescent staining of c-fos (red) and c-jun (red) in UMSCC38 cells transfected with either shGFP or shIGFBP-3. Scale bar is 50 μm. **C.** RT-PCR and western blot analysis of integrin β_4_, c-fos and c-jun expression levels in UMSCC38 cells treated with rBP3 as indicated. **D.** RT-PCR analysis of integrin β_4_, c-fos and c-jun expression levels in stable shGFP- or shIGFBP-3-expressing UMSCC38 cells transfected with either negative control (NC) siRNA or c-jun/c-fos (AP-1) siRNA mixtures. **E.** RT-PCR analysis of integrin β_4_, c-fos and c-jun expression levels in HUVECs treated with IGFBP-3 siRNA and/or c-jun/c-fos siRNAs. To observe the effects of combined knockdown, IGFBP-3 plus c-jun/c-fos siRNAs were co-transfected (BP3 + AP-1); the controls for this group were cells transfected with double the amount of NC siRNA (2× NC).

Next, we attempted to determine how IGFBP-3 regulates integrin β_4_ expression and intracellular effectors of cell adhesion signaling. Cooperation between AP-1 and Ets has been reported to mediate integrin β_4_ promoter activity [28]. Therefore, we investigated the effect of IGFBP-3 on AP-1 activity. To this end, UMSCC38 cells stably transfected with either shGFP or shIGFBP-3 were transfected with a reporter plasmid containing AP-1 binding site. The relative luciferase activity of the AP-1-containing reporter system was increased by the downregulation of IGFBP-3, which was reversed by rBP3 treatment in a dose-dependent manner (Figure 5A). We then investigated the possible impact of IGFBP-3 on the expression of c-jun and c-fos in UMSCC38 cells. Compared to UMSCC38 cells transfected with control shRNA, cells stably transfected with shIGFBP-3 showed increased c-fos and c-jun protein and mRNA expression levels (Figure 5B and 5C). In addition, loss of IGFBP-3 induced the nuclear localization of c-fos and c-jun significantly (Figure 5B). Upon treatment with rBP3, the increase in c-fos and c-jun mRNA levels mediated by loss of IGFBP-3 was reversed significantly, indicating that IGFBP-3 regulates c-fos and c-jun at the transcriptional level (Figure 5C). We then examined whether IGFBP-3 regulates integrin β_4_ expression through AP-1 regulation. We observed that the increased expression levels of c-fos, c-jun, as well as integrin β_4_ in UMSCC38 cells stably transfected with shIGFBP-3 were down-regulated by silencing of c-fos and c-jun expression by siRNA transfection (Figure 5D). AP-1-mediated integrin β_4_ regulation of IGFBP-3 was also confirmed in HUVECs (Figure 5E). These findings indicated that IGFBP-3 regulates integrin β_4_ expression by inhibiting the transcription of c-fos and c-jun and thus suppressing the activity of AP-1 transcription factor.

### IGFBP-3 inhibits AP-1 transcription in both IGF-dependent and IGF-independent manner

**Figure 6:**
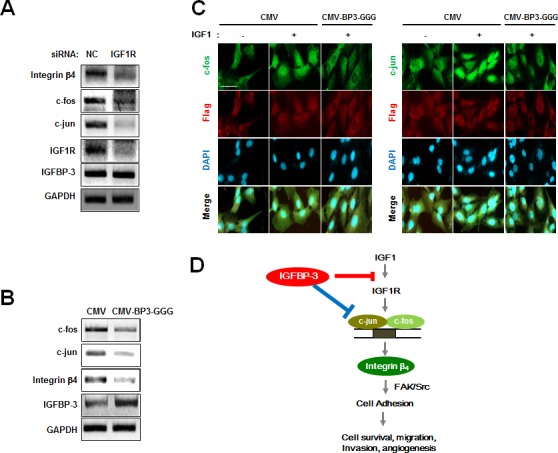
IGFBP-3 inhibits AP-1 transcription in both IGF-dependent and IGF-independent manner **A.** RT-PCR analysis of integrin β_4_, c-fos and c-jun mRNA expression levels in UMSCC38 cells transfected with either NC siRNA or IGF1R siRNA. **B.** RT-PCR analysis of integrin β_4_, c-fos and c-jun expression levels in stable shIGFBP-3-expressing UMSCC38 cells transfected with either CMV or CMV-BP3-GGG. **C.** Immunofluorescent staining of c-fos (green) and c-jun (green) in UMSCC38 cells transfected with either CMV plasmid or CMV-BP3-GGG plasmid and then stimulated with IGF1 (100 ng/ml) for 15 min. Scale bar is 20 μm. **D.** A model of the proposed molecular mechanism for the role of IGFBP-3 in cell adhesion during angiogenesis.

We next assessed whether IGFBP-3-mediated regulation of c-fos and c-jun expression is IGF-independent or -dependent or not. To this end, UMSCC38 cells were transfected with IGF1R siRNA, in which IGF1R silencing significantly decreased expression levels of c-jun and c-fos (Figure 6A). These findings suggest that IGFBP-3 inhibits intracellular cell adhesion signaling by regulating the expression of AP-1, through IGF-dependent mechanisms. Also, we assessed whether IGF-independent mechanisms are involved in the IGFBP-3-mediated regulation of AP-1 expression by using IGFBP-3-GGG. We observed that the increased expression levels of c-fos, c-jun, as well as integrin β_4_ in UMSCC38 cells stably transfected with shIGFBP-3 were down-regulated by IGFBP-3-GGG overexpression (Figure 6B). To confirm this, we assessed the effect of IGFBP-3-GGG on IGF-1-induced AP-1 expression and localization. IGF-1 clearly increased both expression and nuclear localization of c-jun and c-fos, whereas the overexpression of IGFBP-3-GGG blocked both expression and nuclear localization of c-jun and c-fos mediated by IGF-1 (Figure 6C). These findings indicated that IGFBP-3 regulates integrin β_4_ expression by inhibiting the transcription of c-fos and c-jun and thus suppressing the activity of AP-1 transcription factor through IGF-independent mechanism. Collectively, these results suggest that IGFBP-3 may inhibit integrin β4 expression through AP-1 transcriptional regulation in both IGF-dependent and -independent manner.

## DISCUSSION

In this article, we have demonstrated the inhibitory effects of IGFBP-3 on cell-to-matrix adhesion via IGF-dependent and IGF-independent suppression of the expression of a subset of integrins, especially integrin β_4_. Our key findings include the following: 1) the modulation of HNSCC cell (UMSCC38)-to-matrix and vascular endothelial cell (HUVEC)-to-matrix adhesion by gain or loss of IGFBP-3 expression; 2) the capacity of IGFBP-3 to induce both IGF-dependent and IGF-independent inhibition of integrin β_4_ expression; 3) IGFBP-3-mediated disruption of established focal adhesions and actin stress fibers and suppression of FAK and Src phosphorylation; and 4) IGFBP-3-induced blockade of c-jun and c-fos transcription, resulting in inactivation of AP-1 and suppression of AP-1-mediated integrin β_4_ transcription (Figure 6D). Our present results indicate the role of IGFBP-3 in cell adhesion, providing an important proof-of-principle for the development of IGFBP-3 as an anti-adhesive antitumor agent.

During tumorigenesis, neoplastic transformed cells exhibit altered adhesion, adhesion-dependent signaling pathway, and cytoskeletal reorganization, in which the expression pattern and cellular distribution of integrin subunits may be altered and affect ligand binding affinity and transformed cell phenotypes [29, 30]. Furthermore, differentially expressed integrins are associated with tumor growth, metastasis, and angiogenesis as well as cross-talk between tumor cells and their microenvironment [31, 32]. Considering the critical role of integrins in tumor progression, extensive efforts are required for the clinical management of patients with tumors exhibiting high levels of integrin expression. Thus, integrins could be an attractive target for anticancer therapy. Indeed, preclinical and clinical development of integrin inhibitors is ongoing [33, 34]. The antitumor activities of cilengitide, a cyclized Arg-Gly-Glu (RGD)-containing pentapeptide that selectively blocks the activation of the integrins α_v_β_3_ and α_v_β_5_ [35], have been confirmed in various preclinical studies [36-38]. Phase I and II clinical studies have recently evaluated cilengitide in patients with solid tumors, with promising results [39-43].

Accumulating evidence indicates that IGFBP-3, a major IGFBP that modulates the bioavailabilities of both IGF-1 and IGF-2, can induce antitumor activities through IGF-dependent as well as IGF-independent mechanisms [44]. We previously demonstrated that IGFBP-3 inhibits the growth of NSCLC cells *in vitro* and *in vivo via* the PKB/Akt and MAPK signaling pathways, which are activated by IGF-mediated signaling pathways and play important roles in cell survival [10]. We also reported that IGFBP-3 can induce IGF-independent antiangiogenic activities by directly interacting with Erk1/2 and thus inactivating Erk1/2 and Elk-1, leading to the suppression of early growth response protein-1 (Egr-1)-mediated transcriptional activation of basic fibroblast growth factor (bFGF) and platelet-derived growth (PDGF) [8]. However, the effects of IGFBP-3 on cell-matrix adhesion, a critical biophysical parameter that affects cell movement during cancer progression, remain unclear.

Because IGFBP-3 can inhibit cancer progression by inhibiting angiogenesis and the metastatic activities of cancer cells [9], IGFBP-3 was expected to affect cancer and/or vascular endothelial cell-matrix adhesion. In support of this notion, the overexpression of IGFBP-3 *via* adenoviral infection significantly suppresses NSCLC cell adhesion to ECM components, including collagen, fibronectin, and laminin [19]. Consistent with this previous finding, in the current study, modulation of IGFBP-3 *via* treatment with recombinant protein or transfection with expression vectors affected the matrix adhesion of HNSCC cells and HUVECs. This is particularly important because adhesion to the matrix promotes the survival, migration, and invasion of cancer and vascular endothelial cells [45].

While investigating the mechanisms that mediate the antiadhesive activities of IGFBP-3, we observed that IGFBP-3 regulated integrin α_3_, β_1_ and β_4_ (Figure 2B and 2C) as well as the phosphorylation of FAK-Src. Furthermore, we found that integrin α_3_ and β_1_ were also regulated by IGFBP-3 in a similar fashion (Supplementary Figure 2). Integrin β_1_ has been reported to play an important role in tumor initiation, reversion, survival, tumor progression, and metastasis [46-48]. Integrin β_1_ inhibitors were found to achieve good responses in the treatment of refractory tumors and advanced metastatic disease, and inhibitory antibodies (e.g., AIIB2) enhanced radiotherapy efficacy in human breast cancer cells *in vitro* and *in vivo* [49]. Several recent studies have demonstrated that integrin α_3_β_1_ plays an important role in cell transformation, migration, invasion, angiogenesis, and tumor progression [50, 51]. In the complete absence of α_v_β_3_, integrin α_3_β_1_ triggers signals necessary for angiogenesis and tumorigenesis [52]. In addition, efficient skin tumor development is critically dependent on the presence of integrin α_3_β_1_ [53]. Integrin β_4_, an adhesion receptor for basement membrane laminin, is frequently expressed in the endothelium throughout the body [54]. Integrin β_4_ stabilizes and stimulates the formation of actin-rich protrusions in carcinoma cells, which results in tumor invasion [55, 56]. Although the role of integrin β_4_ in the endothelium is not fully understood, integrin β_4_ is regarded as a proangiogenic molecule [55, 56]. These previous findings and the ability of IGFBP-3 to regulate the expression of integrins, including α_3,_ β_1,_ and β_4_, indicate that IGFBP-3 would play a major role in cancer development and progression.

Because inhibition of IGF-IR suppresses the adhesion, invasion, and metastasis of various cancer cell types, we reasoned that the inhibitory effect of IGFBP-3 on integrin expression and cell-matrix adhesion may occur through IGF-dependent mechanisms. Indeed, truncated IGF-1R was secreted extracellularly and inhibited the IGF-1-mediated increase in integrin expression and intracellular cell adhesion signaling. However, the inhibitory effects of IGFBP-3 on integrin expression appear to include novel IGF-independent mechanisms for the following reasons: 1) IGFBP-3 suppresses the expression of integrins and the phosphorylation of FAK and Src induced by a mutant recombinant IGF-1 that is deficient in IGF-1 binding capacity; and 2) the non-IGF-binding IGFBP-3-GGG blocks the effect of IGF-1 on integrin expression and FAK and Src phosphorylation. There are some explanations for the IGF-independent role of IGFBP-3, in which IGFBP-3 interacts with target molecules such as TGF-beta receptor (TβR-V), vitamin D receptor (VDR), and NF-κB [57-59]. Various types of cancer cells and smooth muscle cells have been suggested to possess IGFBP-3 receptors other than IGF-IR, such as TβR-V [57]. These results and our data suggest that the IGFBP-3-mediated suppression of integrin β_4_ expression may be achieved, at least in part, through IGF-independent mechanisms. In this study, we demonstrated that IGFBP-3 regulates AP-1, an important transcription factor for integrin β_4_ expression, through IGF-independent down-regulation of c-Jun and c-Fos transcription. Notably, the UCSC Genome Browser predicted potential binding sites for AP-1 in human integrin β_1_ promoter (data not shown). Therefore, additional is required to determine whether IGFBP-3 mediate the integrin β_1_ through AP-1 regulation.

In summary, our data provide experimental evidence for the antiadhesive activity of IGFBP-3 through the suppression of integrin β_4_ expression and, consequently, its downstream signaling cascade. Given that the clinical development of integrin inhibitors is ongoing [39-43], the IGF-independent inhibitory actions of IGFBP-3 in regulating integrin β_4_ expression and integrin β_4_-FAK-Src signaling suggest that IGFBP-3 represents a promising antineoplastic agent. These findings warrant clinical trials to evaluate the therapeutic value of IGFBP-3 treatment. Further studies are also warranted to understand the precise molecular mechanism of IGFBP-3-mediated regulation of integrin β_4_.

## MATERIALS AND METHODS

### Antibodies and reagents

Anti-integrin antibodies were purchased from BD Biosciences (San Diego, CA), and the subunit clones were as follows: anti-α_2_; anti-α_3_; anti-α_5_; anti-αv; anti-β_1_; anti-β_3_; and anti-β_4_. Anti-phospho-focal adhesion kinase (FAK) (Tyr-397), anti-total-FAK, anti-phospho-c-Src (Tyr-416), and anti-c-Src antibodies were obtained from BioSource International (Camarillo, CA). Anti-phospho-IGF-1R (Tyr-1131), anti-total IGF-1R, anti-phospho p44/p42 MAP kinase (Thr-202/Tyr-204) anti-total p44/p42 MAP kinase, anti-phospho AKT (Ser-473) and anti-total AKT were purchased from Cell Signaling Technology and Santa Cruz Biotechnology. For the indirect immunofluorescence studies, Alexa 488-conjugated IgG and Alexa 546-conjugated IgG were obtained from Molecular Probes. For the western blot analyses, horseradish peroxidase-linked anti-mouse and anti-rabbit IgG were purchased from Amersham Life Sciences (Arlington, IL). Adenoviruses expressing wild type IGFBP-3 (Ad-BP3) or mutant IGFBP-3 (Ad-BP3-GGG) were established using plasmids encoding Flag-BP3-wt and Flag-BP3-GGG [27]. Empty virus (Ad-EV) was used as a negative control. Bovine serum albumin and gelatin were obtained from Sigma-Aldrich (St. Louis, MO, USA). Fibronectin was purchased from Invitrogen, and type I collagen and laminin were obtained from BD Biosciences. Recombinant human IGF-I and IGFBP-3 (rBP3) were purchased from R&D Systems (Minneapolis, MN, USA). For treatment, we purchased recombinant human mutant IGF-I (Mut IGF-1) (Upstate Biotech, NY), which is resistant to IGFBP-3 for 25 min.

### Cell culture

UMSCC38 (HNSCC cell lines) and HUVECs (Clonetics, San Diego, CA) were cultured as described previously [8]. HUVECs were cultured in endothelial cell basal medium (EBM-2; Clonetics) supplemented with EGM-2 SingleQuots (Clonetics). Cells between passages 3 and 8 were used.

### Transient and stable cell transfection

HUVECs were transiently transfected with 5 μg of pCMV6-EV (CMV), pCMV6-IGFBP-3-Flag (CMV-BP3), or pCMV6-IGFBP-3-ggg-Flag (CMV-BP3-ggg) [8]. After serum starvation, the cells were stimulated with 10% FBS for 20 min. To prepare stable cell lines, UMSCC38 cells were transfected with pSM2 retroviral vectors containing a short hairpin small interfering RNA against human IGFBP-3 or a control GFP vector under the control of the U6 promoter (Open Biosystems). Stable cell lines were selected with 0.8 μg/mL puromycin and screened for IGFBP-3 protein expression.

### Cell adhesion assay

Each well of a 96-well culture plate was coated with type I collagen (10 μg/ml), laminin (20 μg/ml), gelatin (5 μg/ml), and fibronectin (10 μg/ml), followed by incubation overnight at 4°C. After washing with phosphate-buffered saline (PBS), 3.0% BSA was added to each well for 1 h to prevent nonspecific attachment. Suspended cells in serum-free media were added to each coated well. After incubation at 37°C for 30 min, non-adherent cells were removed by streaming PBS over the plate 3 times. The remaining adherent cells were stained with 0.2% crystal violet and washed with PBS several times to remove excess dye. The stained crystal violet was dissolved in DMSO and measured by scanning with an ELISA reader (Tecan) with a 590-nm filter. Each sample was assayed in triplicate, and the experiment was repeated twice independently.

### Immunoblotting

Total cellular extracts were separated by SDS-PAGE in 8-12% gels, transferred onto 0.2-μm nitrocellulose membranes (Schleicher and Schuell, Dassel, Germany), blocked with 3% BSA in PBS containing 0.1% Tween-20 and incubated with the appropriate primary antibodies overnight at 4°C. Immunoreactive bands were visualized using peroxidase-conjugated secondary antibodies and an ECL western blot detection system (Amersham).

### Immunofluorescent staining

HUVECs were seeded on glass coverslips and cultured to confluence. After treatment, cells were washed twice with cold PBS, fixed in 4% paraformaldehyde for 10 min, permeabilized with 0.1% Triton X-100 for 15 min, and blocked with 10% normal goat serum for 30 min. Cells were incubated with primary antibody overnight at 4°C, washed three times with PBS, and incubated for 50 min with Alexa 546-conjugated or Alexa 488-conjugated IgG (Molecular Probes) at a 1:1000 dilution as the secondary antibody. Nuclei were stained with 4′-6-diamidino-2-phenylindole (DAPI, Invitrogen). Images were acquired with an LSM 710 microscope (Zeiss).

### Agilent human genome 8×60K microarray processing and hybridization

The microarray experiments were designed to study the effect of IGF-BP3 on UMSCC38 cells stably transfected with either shGFP RNA or shIGFPBP-3 RNA. mRNA was purified and hybridized and microarrays were scanned according to the manufacturer's protocols (Agilent Technologies). Quantification was performed with the GeneSpring GX v.11.5.1 software, which allows multifilter comparisons using data from different experiments to perform normalization, list generation, and functional classification of differentially expressed genes. The expression of each gene was reported as the ratio of the value obtained after each condition relative to the control condition after normalization and statistical analysis of the data. A corrected cutoff value of <0.05 was applied, and the output of this statistical analysis was filtered by fold expression to specifically select differentially expressed genes.

### Statistical analysis

Data are presented as the mean ± standard deviation (SD) and were analyzed using two-tailed t-tests. P<0.05 was considered statistically significant.

## SUPPLEMENTARY MATERIAL FIGURES


