# Supplementary material for: Insulin-like growth factor binding protein-3 inhibits cell adhesion via suppression of integrin β4 expression
Source: Oncotarget. 2015 Apr 14;6(17):15150–63. doi: 10.18632/oncotarget.3825 (PMC4558142; doi:10.18632/oncotarget.3825)
Supplement: Supplementary file 1 [file oncotarget-06-15150-s001.pdf]

Insulin-like growth factor binding protein-3 inhibits cell adhesion via suppression of integrin β4 expression

Supplementary Material

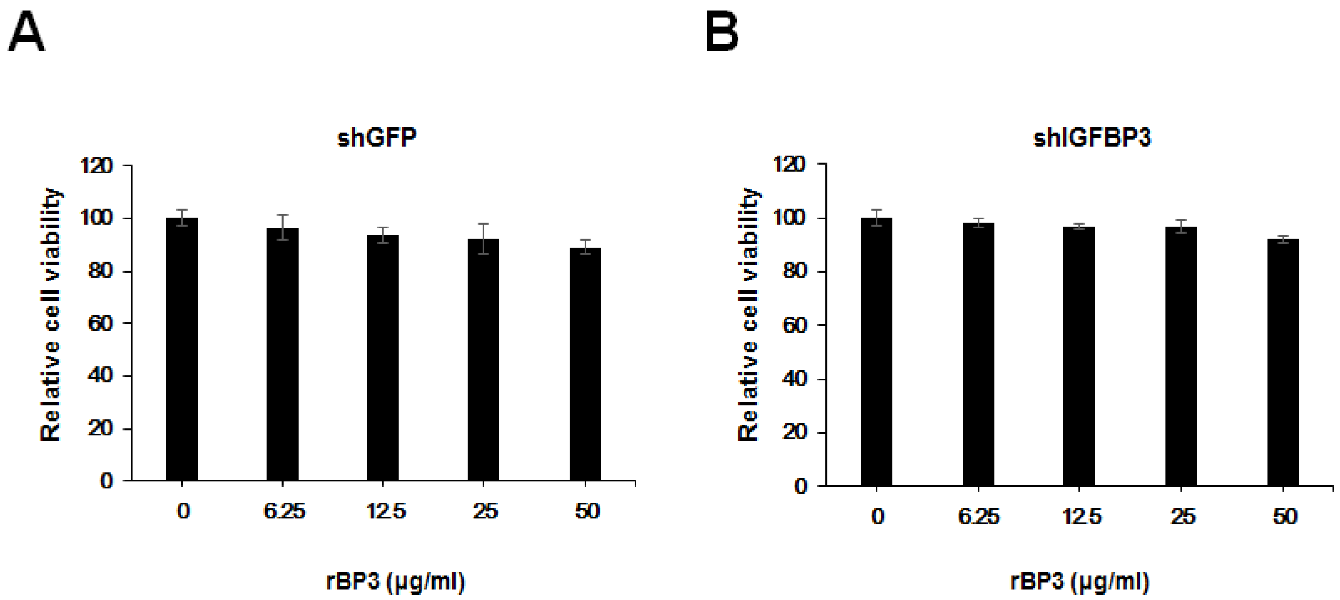

Supplementary Figure 1: IGFBP-3 did not affect cell viability of UMSCC38 cells for 3days. UMSCC38 cells were stably transfected with retroviral pSM2 plasmids ((A) control shGFP RNA (shGFP) or (B) the shIGFBP-3 RNA (shIGFBP-3)) and treated with recombinant IGFBP-3 (rBP3) for 3 days. Cell proliferation was assessed by MTT assay. Cell viability values are expressed as relative to non-treated cells for 3 days, normalized to 100%.

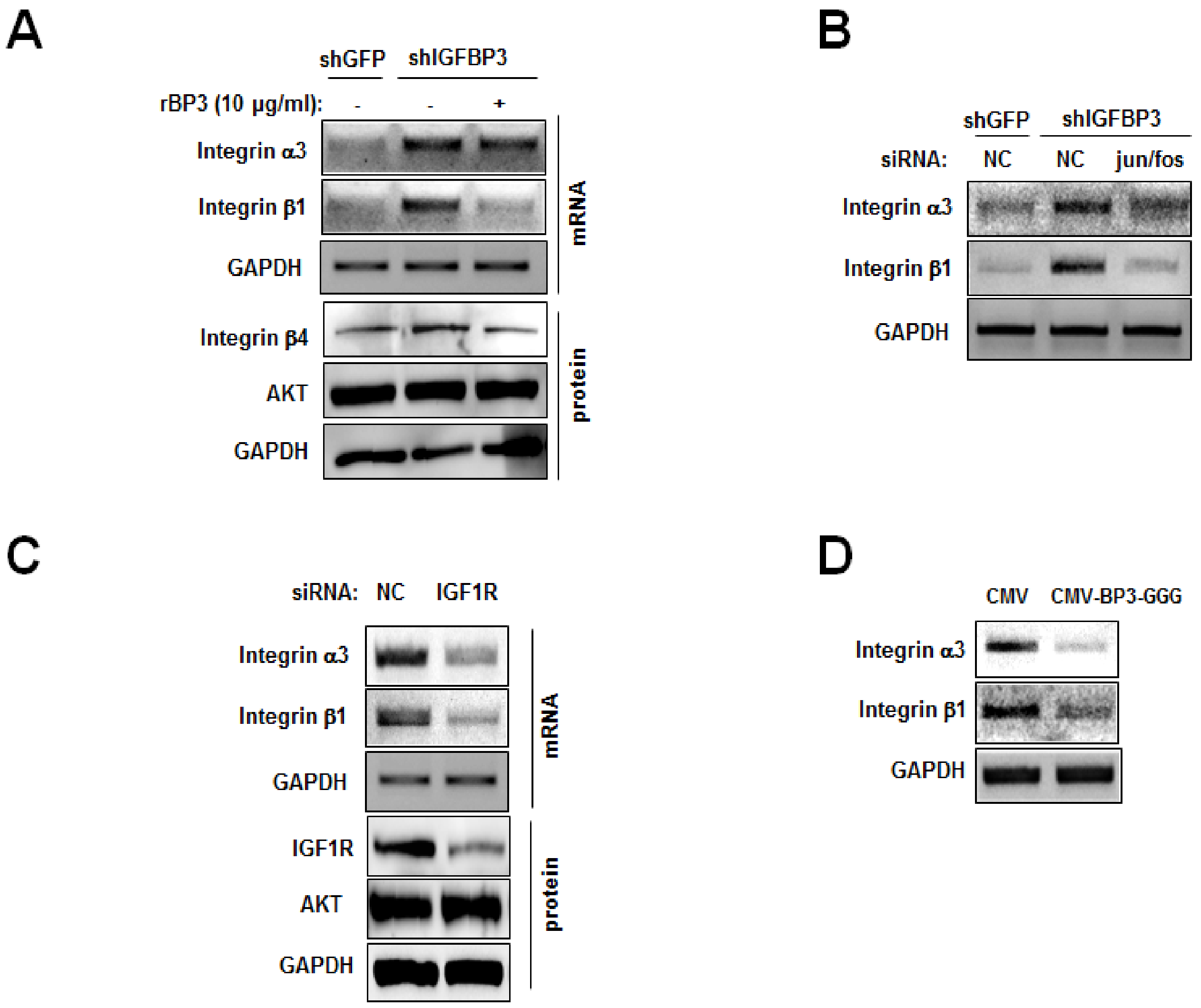

Supplementary Figure 2: IGFBP-3 reduces integrin α3 and integrin β1 expression in UMSCC38 cells. (A) UMSCC38 cells were stably transfected with retroviral pSM2 plasmids [control shGFP RNA (shGFP) or the shIGFBP-3 RNA (shIGFBP-3)] and treated with recombinant IGFBP-3 (rBP3). The expression levels of integrin α3 and integrin β1 were assessed by RT-PCR assay (top). The expression levels of integrin β4 and AKT were assessed by Western blot assay (bottom). (B) UMSCC38 cells were stably transfected with retroviral pSM2 plasmids [control shGFP RNA (shGFP) or the shIGFBP-3 RNA (shIGFBP-3)] and transfected with either control siRNA or AP-1 siRNA. The expression levels of integrin α3 and integrin β1 were assessed by RT-PCR assay. (C) UMSCC38 cells were transfected with either control siRNA or IGF1R siRNA. The expression levels of integrin α3 and integrin β1 were assessed by RT-PCR assay (top). The expression levels of integrin β4 and AKT were assessed by Western blot assay (bottom). (D) UMSCC38 cells were transfected with either pCMV6-empty vector (CMV) or pCMV6-IGFBP-3-GGG (CMV-BP3-GGG). The expression levels of integrin α3 and integrin β1 were assessed by RT-PCR assay.
